# Hypoxia promotes acquisition of aggressive phenotypes in human malignant mesothelioma

**DOI:** 10.1186/s12885-018-4720-z

**Published:** 2018-08-15

**Authors:** Myung-Chul Kim, Sung-Hyun Hwang, Na-Yon Kim, Hong-Seok Lee, Sumin Ji, Yeseul Yang, Yongbaek Kim

**Affiliations:** 1 Laboratory of Clinical Pathology, College of Veterinary Medicine, Seoul National University, 1 Gwanak-ro, Gwanak-gu, Seoul 08826 South Korea; 2 BK21 PLUS Program for Creative Veterinary Science Research, College of Veterinary Medicine, Seoul National University, 1 Gwanak-ro, Gwanak-gu, Seoul 08826 South Korea; 3 Research Institute for Veterinary Science, College of Veterinary Medicine, Seoul National University, 1 Gwanak-ro, Gwanak-gu, Seoul 08826 South Korea

**Keywords:** Mesothelioma, Hypoxia, Tumor microenvironment, Malignant phenotypes, HIFα, Stemness, EMT, Drug resistance

## Abstract

**Background:**

Hypoxia is a hallmark of the solid tumor microenvironment and is associated with poor outcomes in cancer patients. The present study was performed to investigate mechanisms underlying the hypoxia-induced phenotypic changes using human malignant mesothelioma (HMM) cells.

**Methods:**

Hypoxic conditions were achieved by incubating HMM cells in the air chamber. The effect of hypoxia on phenotype changes in HMM cells was investigated by performing in vitro clonogenicity, drug resistance, migration, and invasion assays. Signaling pathways and molecules involved in the more aggressive behaviors of HMM cells under hypoxia were investigated. A two-tailed unpaired Student’s *t*-test or one-way ANOVA with Bonferroni post-test correction was used in this study.

**Results:**

Hypoxic conditions upregulated hypoxia-inducible factor 1 alpha (HIF-1α) and HIF-2α in parallel with the upregulation of its target, Glut-1, in HMM cells. In vitro clonogenicity of HMM cells was significantly increased in hypoxic conditions, but the proliferation of cells at a high density in hypoxia was lower than that in normoxic conditions. The expression levels of HIF-2α and Oct4 were increased in hypoxic HMM cells. The percentage of cells with high CD44 expression was significantly higher in HMM cells cultured in hypoxia than those cultured in normoxia. Hypoxia significantly enhanced the resistance of HMM cells to cisplatin, which occurred through cytoprotection against cisplatin-induced apoptosis. While cisplatin treatment decreased the ratio of Bcl-2 to Bax in normoxic condition, hypoxia conversely increased the ratio in HMM cells treated with cisplatin. Hypoxia increased the mobility and invasiveness of HMM cells. Epithelial to mesenchymal transition was promoted, which was indicated by the repression of E-cadherin and the concomitant increase of vimentin in HMM cells.

**Conclusions:**

The data illustrated that hypoxic conditions augmented the aggressive phenotypes of HMM cells at the biological and molecular levels. The present study provides valuable background information beginning to understand aggressiveness of HMM in tumor microenvironments, suggesting that a control measure for tumor hypoxia may be an effective therapeutic strategy to reduce the aggressiveness of cancer cells in HMM patients.

**Electronic supplementary material:**

The online version of this article (10.1186/s12885-018-4720-z) contains supplementary material, which is available to authorized users.

## Background

Hypoxia is a common feature of tumor microenvironment [1]. There are two types of hypoxia in solid tumors, intermittent hypoxia and chronic hypoxia. Intermittent hypoxia results from abnormal blood flow associated with transient fluctuations in tumor perfusion and the high permeability of tumor vessels with interstitial hypertension [2]. Chronic hypoxia arises due to the inability of the vascular system to supply the growing tumor mass with adequate amounts of oxygen [3]. Both types of tumor hypoxia have been reported to be correlated with poor outcomes in many cancer patients [4]. Hypoxia enhances cancer cell survival, metastasis, and drug resistance in multiple tumor types [1, 4].

One of the adaptive cellular responses to hypoxia is to increase the expression of hypoxia-inducible factor alpha (HIFα), a subunit of the heterodimeric transcription factor HIF [5]. In normoxia, the HIFα subunit is hydroxylated by prolyl hydroxylases (PHD) and recognized by an E3 ubiquitin ligase, von Hippel-Lindau protein, which proteasomally degrades the HIFα protein [6]. Under hypoxia, however, HIFα becomes stable and starts to accumulate in cancer cells by blocking the von Hip-mediated ubiquitin-proteasome pathway [6]. HIFα heterodimerizes with HIF-1β and migrates to the nucleus. The heterodimer recognizes and binds the hypoxia-responsive element located in the promotor of hundreds of genes [6]. Transcriptional activity of HIF in cancer cells is largely mediated by HIF-1α and HIF-2α [7]. The function of HIF-1α has been extensively investigated in nearly every stage of tumor progression [8]. Recently, growing evidence has suggested that HIF-2α is also a critical mediator of aggressive cancer phenotypes including metastasis and dedifferentiation [9]. Although HIF-1α and HIF-2α activate numerous hypoxia-induced genes harboring HIF binding motifs, each HIFα subunit has its own preferential targets. For example, HIF-1α induces genes primarily involved in anaerobic glycolysis, angiogenesis, and apoptosis [3, 8]. On the other hand, HIF-2α regulates genes that promote invasion and stemness [9, 10]. Depending on tumor types or hypoxic duration, the HIFα isoforms are mutually cooperative or exclusive for biological functions and phenotypes [7].

Human malignant mesothelioma (HMM) is an aggressive malignancy arising from the mesothelium on the surface of the body cavity [11]. Exposure to asbestos fibers increases the risk of HMM, but simian virus 40 may also have a role in HMM tumorigenesis [11]. The occurrence rate of HMM is anticipated to increase worldwide [12]. HMM is highly resistant to traditional anticancer drugs [13]. Several mechanisms of drug resistance have been proposed in HMM, including drug transporters, anti-apoptosis, and antioxidant defenses [14]. Despite the advances in systemic chemotherapy using an antifolate-platinum regimen have improved clinical outcomes in HMM patients, overall prognosis remains poor with median survival times of 4 to 13 months from the initial diagnosis [15, 16].

The existence of hypoxic cells within a tumor is associated with modulation of the malignant process in many cancers [1, 5]. Recent studies have revealed that HMM contains hypoxic regions, suggesting a potential link between tumor hypoxia and ineffective therapeutic efficacy [17, 18]. However, the mechanism underlying the effect of hypoxia on HMM remains largely unknown. The present study showed that hypoxia promotes aggressive phenotypes of HMM cells. Hypoxia enhances in vitro clonogenicity, migration, invasion, and drug resistance through inhibition of apoptosis in HMM cells. Various signaling networks and molecular candidates were suggested for the aggressive biological behaviors of HMM cells, including HIF-1/2α and Oct4 signaling pathways, epithelial to mesenchymal transition (EMT), and Bcl-2 regulation. Exploiting tumor hypoxia may be an alternative therapeutic strategy to reduce the aggressive behavior of HMM cells.

## Methods

### Cell culture and cell lines

MS1 and H513 cell lines were kindly provided by Dr. Jablons (University of California, San Francisco) and Dr. R Kratzke (University of Minnesota), respectively. The cell lines were cultured in RPMI 1640 medium (Mediatech Inc., Manassas, VA, USA) containing 10% fetal bovine serum (FBS; Mediatech Inc.), 10 mM of glucose, 10 mM of HEPES (Sigma-Aldrich, St. Louis, MO, USA), 1.5 g/L sodium bicarbonate (Sigma-Aldrich), 1 mM of sodium pyruvate (Sigma-Aldrich), and 100 U/100 μg/mL penicillin/streptomycin (Gibco-Life Technology, Gaithersburg, MD, USA) at 37 °C in a humidified atmosphere containing 5% CO_2_. To establish a glucose-starved condition, HMM cells were cultured in DMEM medium (Gibco-Life Technology) supplemented with 0 or 1 mM of D-(+)-glucose (Sigma-Aldrich). The HMM cell lines were determined to be free of mycoplasma contamination by using e-Myco Mycoplasma PCR detection kit (e-Myco, iNtRON Biotechnology, Sungnam, Korea).

### Hypoxic condition

Hypoxia was generated by infusing a pre-analyzed air mixture (2.2% O_2_/5% CO_2_/92.8% N_2_) at a flow rate of 5 L/min for 15 min into an air chamber (Billups-Rothenberg Inc., Del Mar, CA, USA) with inflow and outflow valves. Hypoxic treatment of cells was achieved by incubating the cells in the air chamber maintained in a humidified environment at 37 °C. The culture medium was replaced just before carrying out hypoxic treatment.

### Cell proliferation and cytotoxicity assay

Cell proliferation was determined by counting the number of cells and measuring cell metabolic activity using a 3-(4,5-dimethylthiazol-2-yl)-2,5-diphenyl tetrazolium bromide (MTT) dye in each well at defined intervals. Cells were seeded on 6-well plates or 96-well plates at a different density per well. For cell proliferation by manual counting and MTT assay, each group was replicated in five and six separate wells, respectively. The following day, the cells were subjected to normoxia or hypoxia for the indicated periods either without drugs or with varying concentrations of cisplatin (Dong-A Pharm, Seoul, the Republic of Korea). For cytotoxicity assay, each group was replicated in three separate wells. After the treatment, the cells were enumerated using a hemocytometer under an Olympus CK2 microscope (Optical Co., Ltd., Tokyo, Japan). After incubation in MTT dye (5 mg/mL) for 2 h at 37 °C, protected from the light, the absorbance values were determined at 570 nm by the microplate reader (Gen5, Epoch, Bio Tek, Winooski, VT, USA). The absorbance from untreated control cells under each normoxic and hypoxic condition was considered representative of 100% cell viability. All other measurements were expressed as a percentage of the control cell value ± standard deviation.

### Clonogenicity assay

To prevent cell to cell contact or the overlapping of too many colonies, 200 cells were chosen to seed on 6-well plates, as previously described [19]. Each group was replicated in four separate wells. When attached to the plate, the cells were subjected to incubation for 48 h under normoxia or hypoxia. After the incubation, the culture medium was replaced, and the cells were further incubated at 37 °C for 5 days. The cells were fixed with methanol for 5 min, stained with Diff-Quik solution (Merck, Darmstadt, Germany), and dried. Groups of more than 50 cells were counted as viable colonies. The colony forming ability was determined by calculating the percentage of surviving cells based on the plating efficiency that is a ratio of the number of colonies to the number of cells seeded [19].

### Wound healing assay

HMM cells were seeded on 24-well plates at cell densities of 10^5^ cells per well in triplicate. When the cells reached 90% confluence, the culture medium was replaced with one containing mitomycin C (Sigma-Aldrich) at a final concentration of 2 μg/mL, followed by further incubation for 2 h. Mitomycin C was used to minimize the proliferative effect of cancer cells, and the concentration used in this study was found to be non-cytotoxic [20]. The cell monolayer was manually scratched with a 1000 μl pipette tip. The cells were subjected to further incubation in normoxia or hypoxia for 48 h. The area of the scratch distance was photographed under a phase contrast microscope using an Olympus CK2 camera (Optical Co.) at 0 and 48 h of incubation. Cell migration was determined by measuring the migration distance. The results were normalized to the initial scratch distance and presented as % with the normoxic condition set at 100%.

### Invasion assay

The 24-transwell plates (8.0 μm pore size with poly-carbonate membrane; Corning Costar, Lowell, MA, USA) covered with 2 mg/ml basement membrane Matrigel matrix (BD Biosciences, Bedford, MA, USA) were used for the invasion assay. Each group was replicated in three separate wells. The coated transwell was hydrated with culture medium for 2 h prior to cell seeding. HMM cells were resuspended in serum-free medium and seeded at a cell density of 2.5 × 10^4^ into the upper invasion chamber. Culture medium containing 10% FBS was then added to the lower chamber. The cells were incubated at 37 °C for 24 h under normoxia or hypoxia. After a day of incubation, the cells that had invaded the lower surface of the membrane were fixed with methanol for 5 min, stained with Diff-Quik solution (Merck), and quantified by counting five random fields using a phase contrast microscope. Invasion was expressed as the ratio of invading cells incubated under hypoxia compared to the controls in normoxia.

### Apoptosis assay

HMM cells were seeded on 60 mm^2^ petri dishes with confluency of 60% density in quadruplicate. On the following day, the cells were incubated in normoxia or hypoxia for 48 h either with or without cisplatin (10 μM). After the incubation, apoptosis was evaluated using the Annexin V-FITC apoptosis detection kit (Komabiotech, Inc., Seoul, Korea). Briefly, cells were harvested, washed, and incubated in binding buffer containing a saturating concentration of FITC-conjugated Annexin V in the dark for 15 min at room temperature. After being washed with a binding buffer, the cells were resuspended and incubated with 500 μl of binding buffer containing 10 μl of propidium iodide (PI) on ice. The cells were immediately analyzed for the fluorescence of fluorescein isothiocyanate (FITC) and PI using flow cytometry (Becton Dickinson, San Jose, CA, USA). Cells undergoing early and late apoptosis were determined.

### Cell cycle analysis

HMM cells were plated on 6-well plates at confluency of 50% density in triplicate. After a day of incubation, the cells were subjected to normoxia or hypoxia for 24 and 48 h. At the indicated time points, cells were harvested and fixed with ice-cold 70% ethanol for 2 h at − 20 °C. After being washed with PBS, the cells were incubated with PI/RNase staining buffer (BD Pharmingen, BD Biosciences) for 15 min at room temperature. The cells were immediately subjected to analysis using a flow cytometer (BD Biosciences) for the determination of their DNA contents.

### Measurement of cell surface CD44 expression

HMM cells were plated on 6-well plates in triplicate. After 48 h of hypoxia incubation, HMM cells resuspended in PBS were incubated with primary antibody against CD44 (Genetex, CA, USA) at 4 °C for 1 h in the dark. Secondary antibody goat anti-rat IgG-PE (Santa Cruz, CA, USA) for CD44 analysis was incubated at room temperature for 30 min in the dark. After washing with PBS, HMM cells were resuspended in PBS and subjected to flow cytometric analysis for CD44 expression. Unstained cells were used to gate on live cells. After excluding cell debris from the gated populations, a minimum of 10,000 events per condition were collected for the analysis.

### Western blot analysis

After washing cells with cold PBS twice, cell lysates were obtained using RIPA lysis buffer containing complete protease inhibitors. The same amounts of protein were subjected to sodium dodecyl sulfate-polyacrylamide gel electrophoresis; subsequently, the protein bands were transferred onto a nitrous membrane by a wet transfer apparatus. After blocking with 5% non-fat milk at room temperature for 60 min, the nitrous membrane was placed in 0.1% Tween 20 PBS (T-PBS) containing primary antibodies including HIF-1α (1:1000, Cell Signaling), HIF-2α (1:1000, Cell Signaling), E-cadherin (1:1000, Cell Signaling), Bcl-2 (1:1000, Cell Signaling), Bax (1:1000, Cell Signaling), Bcl-xL (1:1000, Cell Signaling), Oct4 (1:1000, Cell Signaling) and vimentin (1:1000, Cell Signaling) and incubated overnight at 4 °C. An antibody against β-actin (1:1000, Cell Signaling) was used as a loading control. After the blot was washed with T-PBS three times for 10 min each, peroxidase-labeled secondary anti-rabbit or anti-mouse antibodies (1:2000) were applied to the blot for 90 min. The protein levels were detected on CL-Exposure film with the use of enhanced chemiluminescence detection reagents (Advansta, Menlo Park, CA, USA) according to the manufacturer’s instructions. Densitometric analysis was performed using the ImageJ program (http://rsbweb.nih.gov/ij).

### Gene expression and quantitative real-time PCR

Total RNA was isolated from the HMM cell lines using the RNeasy Plus Mini Kit protocol (Qiagen, Valencia, CA, USA). The quality and quantity of the total RNA were assessed using Nanodrop (Nanodrop Technologies, Wilmington, DE, USA). Total RNA of 500 ng was used to synthesize cDNA using QuantiTect Reverse Transcription Kit (Qiagen, Valencia, CA, USA). Quantitative real-time PCR was performed using the Rotor-Gene SYBR Green RT-PCR Kit (Qiagen, Valencia, CA, USA). PCR conditions were as follows: 1 cycle at 95 °C for 10 min, followed by 45 cycles of 95 °C for 10 s, and then 60 °C for 30 s. The expression level of each gene was normalized based on endogenous GAPDH expression. The analysis of relative gene expression was determined according to the 2^−ΔΔ^Ct method, as previously described [21]. The primer sequences used in this study are listed in Additional file 1.

### Statistical analysis

All data were presented as the means ± standard deviation. Statistical analyses were done using Microsoft Excel (Microsoft, Seattle, WA, USA) and SPSS software (IBM, Armonk, NY, USA). *P* values were calculated by a two-tailed unpaired Student’s t-test or one-way ANOVA with Bonferroni post-test correction. The results were confirmed in at least three independent experiments and considered to be statistically significant when P value was less than 0.05. All dataset and the statistical information are listed in Additional file 2.

## Results

### Experimental induction of hypoxia in vitro

**Fig. 1:**
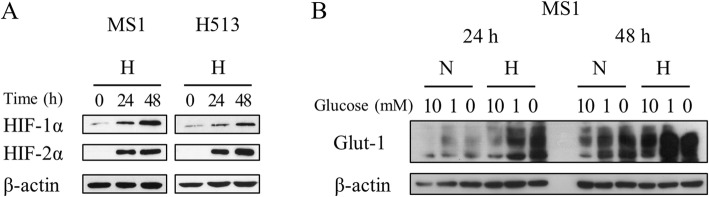
The experimental establishment of tumor hypoxia in HMM cells. (**a**) Hypoxia markedly increased HIF-1α expression and induced HIF-2α expression de novo in HMM cells. (**b**) A HIF-1/2α target Glut-1 increased in response to hypoxia and glucose starvation in MS1 cells. Abbreviations: N, normoxia; H, hypoxia

Experimental establishment of hypoxia was verified by HIFα induction in HMM cells. Western blot analysis confirmed the upregulation of HIF-1α and the de novo synthesis of HIF-2α under hypoxia (Fig. 1a). As hypoxia was prolonged, HIF-1/2α target Glut-1 expression was also elevated, suggesting a functional transcriptional activity of HIF-1α in the hypoxic state (Fig. 1b). Glucose starvation was used as a positive control for Glut-1 expression.

### Hypoxia enhanced in vitro clonogenicity but reduced proliferation of HMM cells

**Fig. 2:**
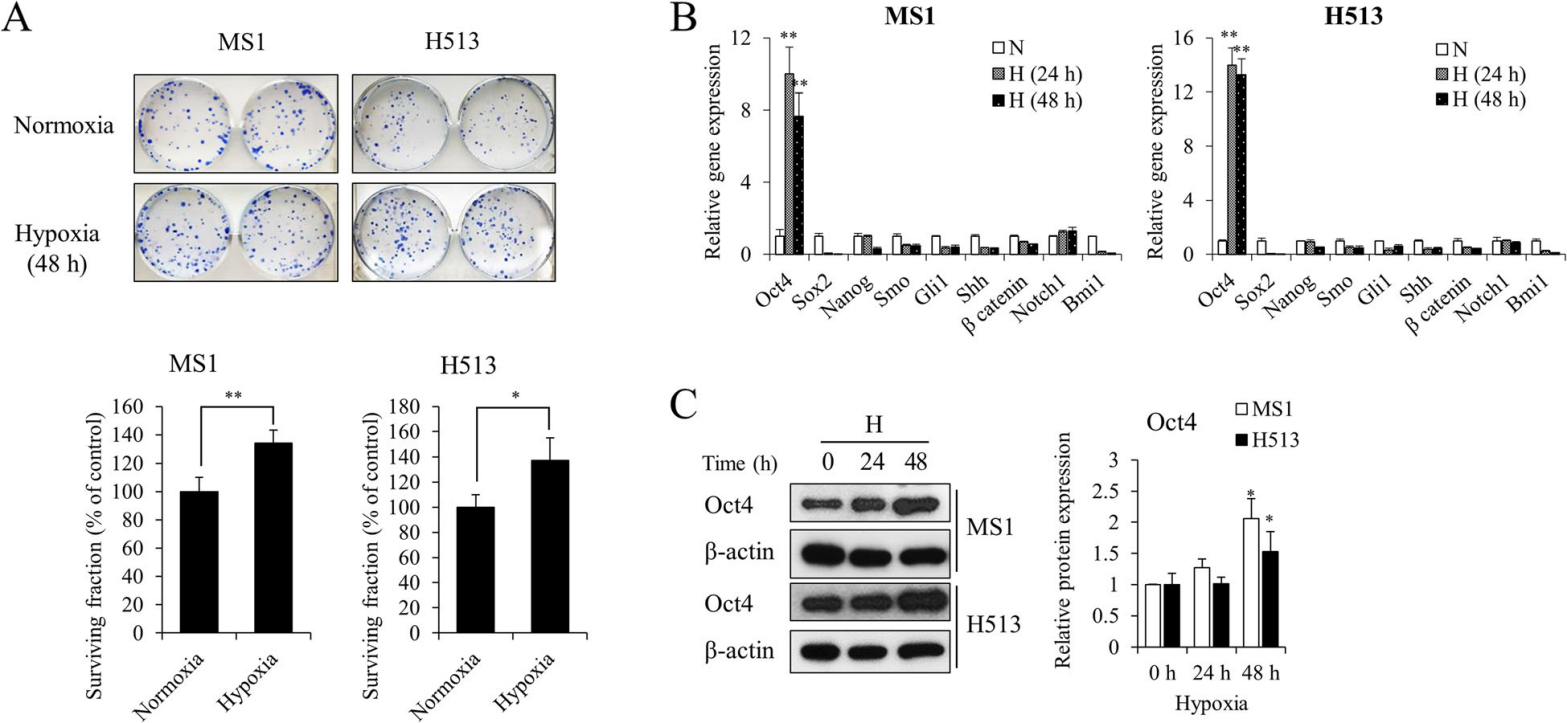
The effect of hypoxia on in vitro clonogenicity in HMM cells. (**a**) Hypoxia enhanced the colony forming ability of HMM cells. Representative microscopic examinations are presented. *P* value was calculated by Student’s *t*-test. Hypoxia significantly upregulated the expression of Oct4 at transcriptional (**b**) and translational (**c**) levels in HMM cells. P value was calculated by one-way ANOVA with Bonferroni post-test. *P value < 0.05, **P value < 0.01. Abbreviations: N, normoxia; H, hypoxia

**Fig. 3:**
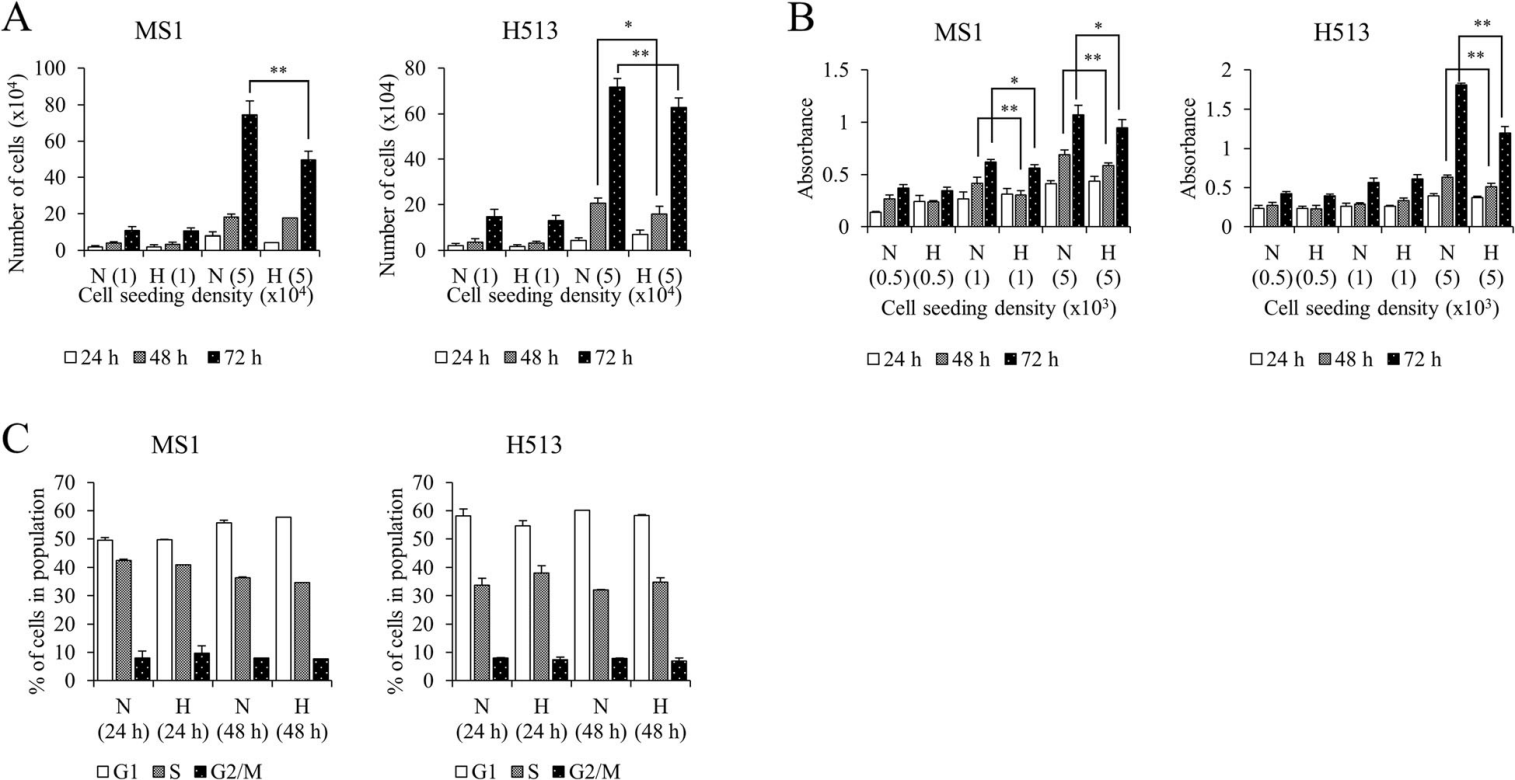
The effect of hypoxia on cell proliferation in HMM cells. Hypoxia significantly decreased proliferation and viability in HMM cells at high cell seeding density. (**a**) Counting cell numbers. (**b**) MTT assay. The number of cells initially seeded is presented in parentheses. Cell cycle profiles did not appreciably differ between normoxic and hypoxic HMM cells (**c**). *P value < 0.05, **P value < 0.01, as calculated by Student’s *t*-test. Abbreviations: N, normoxia; H, hypoxia

The plating efficiency of the untreated control was approximately 0.6 in HMM cells. Hypoxia significantly increased the surviving fraction by 34% and 37% in MS1 and H513 cells, respectively, compared to that of normoxic cells (Fig. 2a). Because the ability of tumor cells to form a single colony is related to the acquisition of stemness properties, the levels of a variety of stemness genes were investigated. Among them, Oct4 gene expression was significantly increased in HMM cells under hypoxia (Fig. 2b). The Oct4 protein was also significantly elevated under hypoxia (Fig. 2c). We also attempted to determine cell surface markers that correlate with stem cell signatures, and hypoxia was found to significantly increase the percentage of HMM cells with the high CD44 expression, a putative marker of cancer stemness of HMM (Additional file 3) [22, 23]. On the other hand, chronic hypoxia did not enhance the proliferative capacity of HMM cells. As the cell density increased, an inhibitory effect of hypoxia on cell growth was detected (Fig. 3a). The parallel measurement using MTT dye also confirmed the significant reduction in cell proliferation of HMM cells under hypoxia. The absorbance-based cell viability was decreased after 48 h of hypoxia from the initial seeding density of 1000 and 5000 in MS1 and H513 cells, respectively (Fig. 3b). The reduced proliferation under hypoxia was not attributable to the cell cycle arrest at the G_1_/_0_ phase (Fig. 3c). The data indicated that hypoxia improved single cell survivability that was mediated through stemness acquisition in HMM cells.

### Hypoxia induced drug resistance in HMM cells

**Fig. 4:**
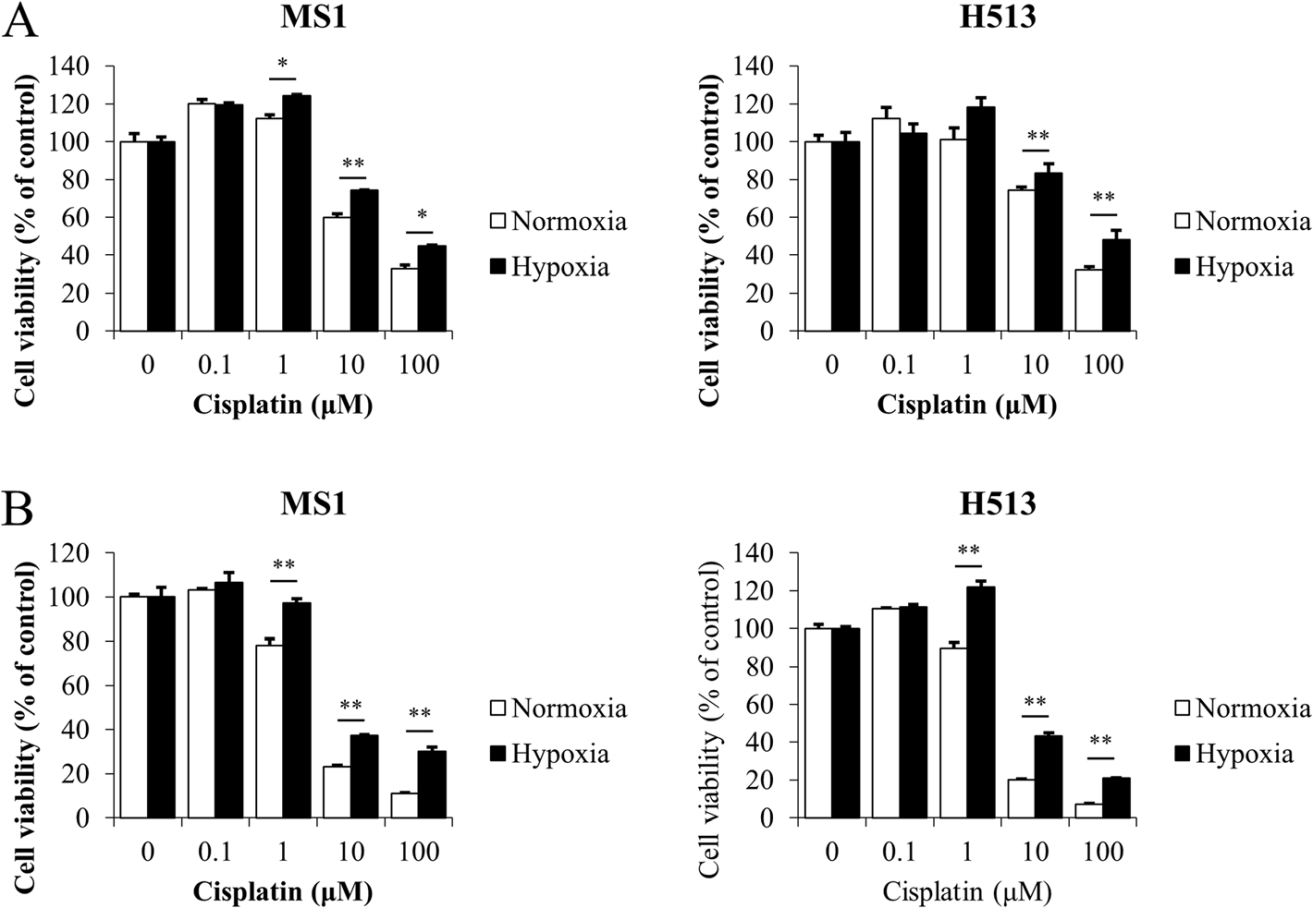
The effect of hypoxia on drug sensitivity in HMM cells. MTT assay. The sensitivity to cisplatin was decreased in HMM cells under hypoxia for 24 h (**a**) and 48 h (**b**), compared to the sensitivity of those in normoxia. * indicates a significant difference compared with the corresponding normoxic cisplatin-treated control. *P value < 0.05, **P value < 0.01, as calculated by Student’s *t*-test

**Fig. 5:**
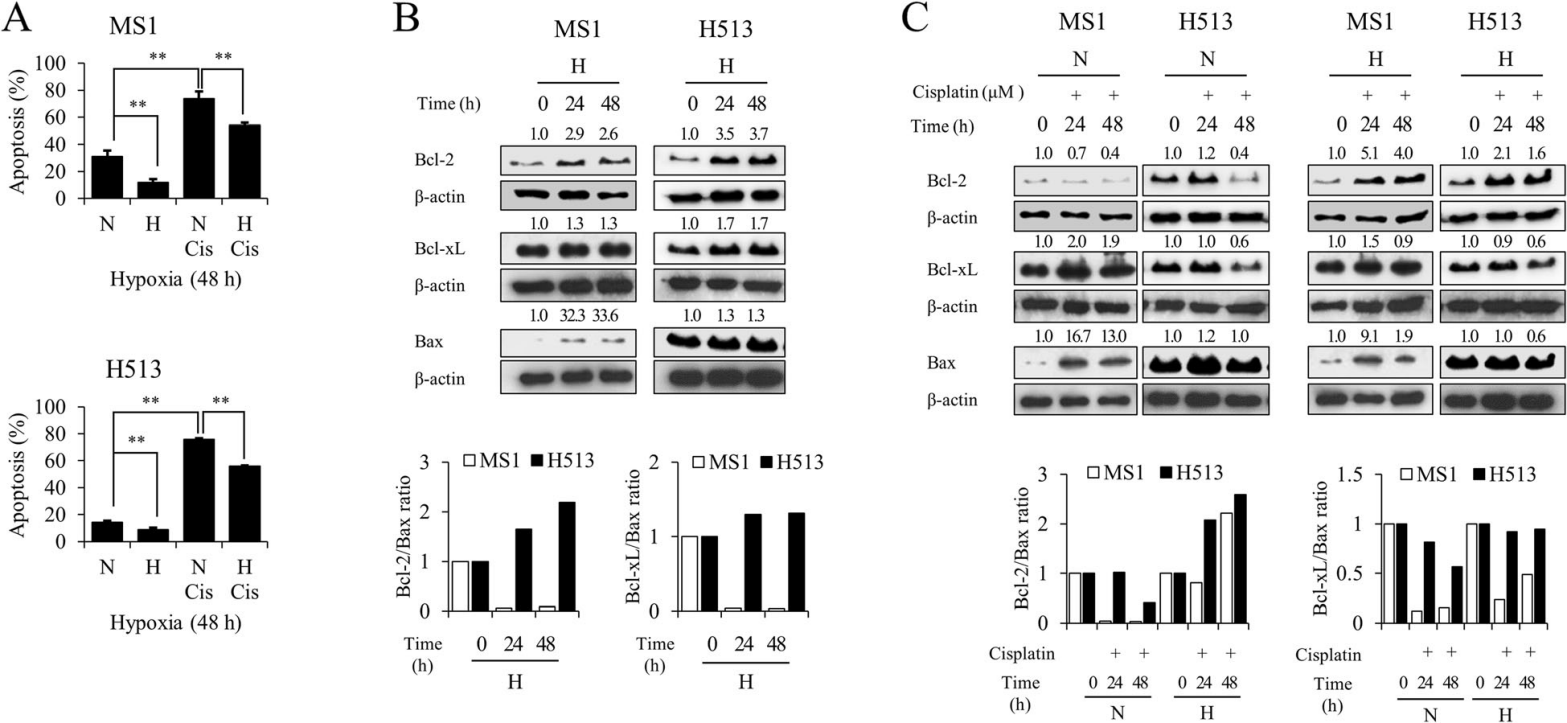
The effect of hypoxia on cisplatin-induced apoptosis in HMM cells. (**a**) Hypoxia significantly reduced apoptosis either with or without cisplatin in HMM cells. (**b**) Hypoxia upregulated the expression of anti-apoptotic Bcl-2, whereas Bcl-xL levels remained almost unchanged in HMM cells. Marked increase in pro-apoptotic Bax expression was detected in MS1 cells only. The ratio of Bcl-2 and Bcl-xL to Bax increased in H513 cells. (**c**) Hypoxia increased Bcl-2 levels and maintained the expression during exposure to cisplatin treatment in HMM cells, compared to the expression level in cells in normoxia. With cisplatin treatment, Bcl-xL expression either tended to decrease or remained unchanged in HMM cells under hypoxia, compared to the level in cells in normoxia. The cisplatin-induced increase in Bax expression was less under hypoxia than under normoxia in MS1 cells. The Bcl-2 to Bax ratio increased in HMM cells under hypoxia following cisplatin. All protein expression was normalized to endogenous β-actin level and is presented as densitometric values at the top of each protein blot. *P value < 0.05, as calculated by one-way ANOVA with Bonferroni post-test

In the normoxic state, cisplatin treatment decreased the cell viability of HMM cells in a dose-dependent manner, but hypoxia significantly reduced the sensitivity of the cells to the drug (Fig. 4). Because apoptosis has been used for the evaluation of the chemotherapeutic efficacy of cisplatin [14], a bivariate Annexin V/PI analysis was performed. Compared to the apoptosis of cells exposed to cisplatin in normoxia, hypoxia not only decreased apoptosis in HMM cells without cisplatin treatment but also significantly inhibited cisplatin-induced apoptosis (Fig. 5a). The expression levels of representative pro- and anti-apoptotic Bcl-2 family members were determined by immunoblot analysis. As shown in Fig. 5b, the Bcl-2 level was increased in HMM cells under the hypoxic state compared to the level in HMM cells under normoxic conditions. However, the level of Bcl-xL remained almost unchanged in hypoxic MS1 cells, and the increase of Bcl-xL was much less in hypoxic H513 cells, compared to that in normoxic H513 cells. A profound elevation of Bax expression was detected in hypoxic MS1 cells, but it remained almost unchanged in H513 cells. Due to the high Bax expression under hypoxia, the ratio of either Bcl-2 or Bcl-xL to Bax was decreased in MS1 cells. The Bcl-2 to Bax ratio remained increased in H513 cells following hypoxia, but the increase in the ratio of Bcl-xL to Bax was not significant. Figure 5c shows the expression profiles of the Bcl-2, Bcl-xL, and Bax in HMM cells treated with cisplatin. In the normoxic state, cisplatin reduced the expression of Bcl-2, but hypoxia increased and maintained the Bcl-2 expression in HMM cells treated with cisplatin. With cisplatin treatment, Bcl-xL expression was decreased in HMM cells as hypoxia was prolonged. The densitometric analysis confirmed the upregulation of Bax expression following cisplatin treatment in MS1 cells. The extent of increase in Bax expression was diminished in hypoxic MS1 cells, compared to the level of MS1 cells in normoxia. While the Bcl-2 to Bax ratio decreased in the normoxic state following cisplatin treatment, hypoxia increased the ratio. The Bcl-xL to Bax ratio was lowered in hypoxic HMM cells treated with cisplatin, compared to that of HMM cells treated with cisplatin in normoxia. Taken together, these results indicated that hypoxia promoted the resistance of HMM cells to cisplatin by regulating Bcl-2 family members.

### Hypoxia enhanced migration, invasion, and epithelial to mesenchymal transition of HMM cells

**Fig. 6:**
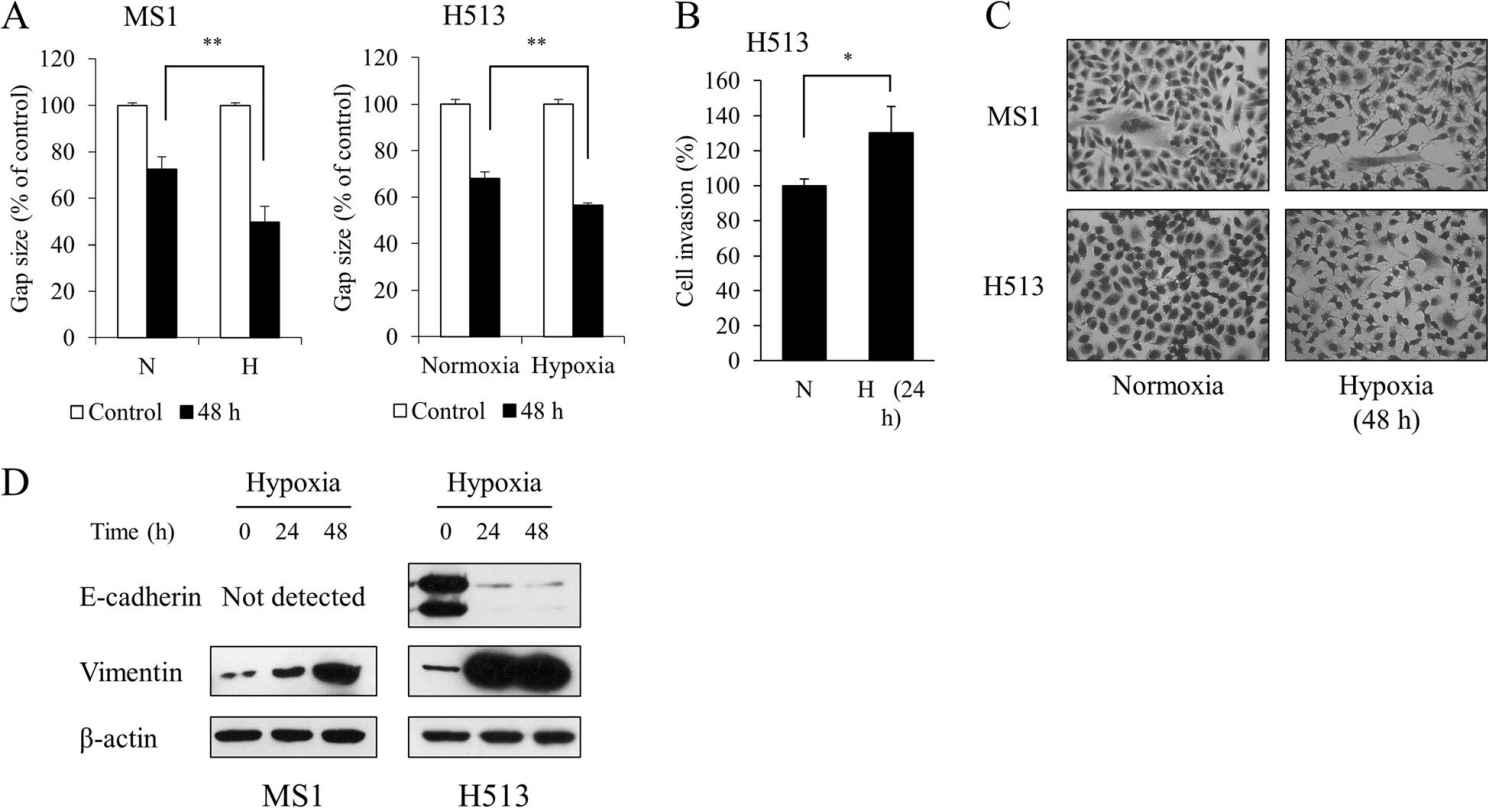
The effect of hypoxia on migration and invasion in HMM cells. (**a**) Hypoxia significantly increased migration in HMM cells. (**b**) Hypoxia significantly enhanced invasion in H513 cells. (**c**) Phase contrast images (400× magnification) of HMM cells cultured for 48 h under normoxia or hypoxia are presented. (**d**) Hypoxia induced a loss of E-cadherin expression and a gain of vimentin expression in HMM cells. E-cadherin expression was not detected in MS1 cells. *P value < 0.05, as calculated by Student’s *t*-test

In the wound healing assay, HMM cells in hypoxia displayed a smaller gap distance than did cells under normoxia (Fig. 6a). Under hypoxia, H513 cells showed increased invasiveness (Fig. 6b). The H513 cells were round to oval or occasionally polygonal with a small amount of cytoplasm, showing high nucleus to cytosol ratio. The MS1 cells were generally spindle to polygonal (Fig. 6c). The HMM cells exposed to hypoxia underwent a morphologic change, showing a neuron-like appearance characterized by pseudopodia protrusions (Fig. 6c). To investigate the mechanisms underlying hypoxia-induced cell migration, the expression levels of two representative EMT-related markers, E-cadherin and vimentin, were analyzed. Western blot analysis revealed that hypoxia reduced the expression of E-cadherin and concomitantly increased the expression of vimentin in HMM cells (Fig. 6d). Vimentin was upregulated in MS1 cells, but E-cadherin was not detected. It might be due to the infrequent expression of E-cadherin in HMM cell lines or primary tumors with mesenchymal cell phenotype [21]. These results showed that hypoxia enhances the acquisition of migratory and invasive phenotypes that are associated with the EMT process in HMM cells.

## Discussion

Tumor cell adaptation to hypoxic condition leads to cancer progression [1, 2, 5]. The present study demonstrates that hypoxia causes HMM cells to behave more aggressively at the biological and molecular levels. Hypoxia enhanced in vitro clonogenicity, migration, invasion, and drug resistance to cisplatin in HMM cells. Various signaling pathways and molecular targets were associated with the hypoxia-induced aggressive behaviors, including HIF-1/2α, EMT, Oct4, and anti-apoptotic Bcl-2. The data presented in this study emphasize the clinical importance of hypoxia in the biology of HMM. Exploiting molecular signaling pathways affected by the hypoxia may help to overcome the low efficacy of traditional anticancer therapy for HMM patients.

In the present study, hypoxia enhanced the in vitro colony forming capability of HMM cells. Bcl-2 antagonization reduces in vitro clonogenicity through apoptosis induction [24, 25]. In this context, the present study showed that the increased Bcl-2 activity likely provided an additional survival advantage to individual HMM cells during colony formation in hypoxia. The effect of hypoxia on the proliferation of HMM cells in culture condition has been reported [26]. Hypoxia enhances tumor stemness by reprogramming non-stem cancer cells to be cancer stem cells (CSCs) with tumor-initiating capacity [27]. The increase of cells with high CD44 expression supports the notion that hypoxia enhanced the stemness of HMM cells [22, 23]. The transformation to CSCs is through the activation of HIFα and stemness-related transcriptional factors, including Oct4, c-Myc, and Notch [27]. In the present study, the enhanced clonogenicity of HMM cells might be associated with increased Oct4 expression, which was mediated through the transcriptional activity of HIF-2α. The upregulation of Oct4 at mRNA level in hypoxic condition contributes to the formation of more viable colonies with different origins of CSCs [28–30]. Heddleston et al. [31] demonstrated the functional significance of HIF-2α and Oct4 in the maintenance of the CSC state under hypoxia.

The weak correlation between Oct4 mRNA and protein expression may suggest the involvement of post-transcriptional modification of Oct4 and/or another stemness factors in enhancing the clonogenicity of HMM cells under hypoxia [32]. CSC factors like c-Myc or Notch are tightly regulated together with Oct4 by the competitive cooperation of HIF-1α and HIF-2α in hypoxic tumor cells [33, 34]. Further studies are warranted to elucidate the functional relationship between HIFαs and stem cell factors including Oct4 in HMM cells. On the other hand, in the present study, once the HMM cells proliferated in high cell populations after completing colony formation, cell growth was decelerated by hypoxia. This phenomenon may be attributable to the insufficient supply of energy required for cellular proliferation under hypoxia. One of the principal actions of HIF-1α is to shift tumor metabolism from oxidative phosphorylation to anaerobic glycolysis [8]. The hypoxic cancer cells consume more glucose by augmenting glucose influx through the upregulation of glucose transporters, such as Glut-1 [1, 5]. Accordingly, Glut-1 overexpression induced by hypoxia suggested the high energy demand or hypoxia-associated nutrient deprivation of HMM cells in this study. Because cell cycle arrest was not determined in hypoxia in this study, the process of protein synthesis could be inhibited, resulting in the decelerated proliferation of HMM cells, as previously suggested [26].

An acquired apoptosis resistance is closely associated with ineffective cancer therapy [3]. Hypoxia inhibits apoptosis in many cancer types following drug treatment [35]. The present study showed that HMM cells acquired a drug-resistant phenotype through inhibition of cisplatin-induced apoptosis under hypoxia. In particular, alteration of Bcl-2 might be responsible for the hypoxia-induced chemoresistance in HMM cells. The Bcl-2 family is a potential blocker of apoptosis and plays a key role in the drug resistance of multiple tumor types [36]. The anti-apoptosis by Bcl-2 is largely due to the inhibition of mitochondrial membrane permeabilization, which can also be activated by pro-apoptotic molecules upon various stimuli that initiate apoptosis [37]. Due to its hydrophobic BH3 binding groove, Bcl-2 engages in multiple anti- and pro-apoptotic Bcl-2 protein-protein interactions, i.e., by binding and sequestering the pro-apoptotic Bax [37]. In this context, the increase of Bcl-2 expression relative to Bax may represent an aspect of hypoxia-induced drug resistance involved in mitochondrial protection upon exposure to cisplatin in HMM cells. In the present study, the role of Bcl-xL in promoting drug resistance under hypoxia is unclear. The inability of H513 cells to induce Bax expression in response to external stimuli may be attributable to the p53 mutation. It has been reported that tumor cells with mutant p53 do not induce Bax expression upon apoptotic stimuli [38]. This is consistent with the previous findings that H513 cells failed to induce Bax expression during TNF-related apoptosis-inducing ligand-mediated apoptosis induced by a histone deacetylase inhibitor [39].

Similar to the previous study [40], hypoxia enhanced the migratory and invasive properties in HMM cells. Additionally, the present study showed that hypoxia promoted EMT process in HMM cells. In HMM, it was shown that the miR-205-mediated reduction of ZEB1 and ZEB2 increased the expression of E-cadherin, which inhibited migration and invasion [41]. The EMT is closely associated with hypoxia and is regulating a more aggressive behavior of cancer cells [42]. In HMM carcinogenesis, HIF-1α stabilization promotes EMT process and stemness via an increased expression of TGF-β and stem cell factors [21]. Moreover, EMT is associated with the emergence of CSCs in HMM cells [43]. Tumor hypoxia is known to recapitulate the HMM microenvironment of the body cavity with HIF-1α expression, and EMT has been potentially implicated in mesothelial carcinogenesis [44, 45]. HIF-1α regulates the expression of a variety of genes involved in EMT-triggering pathways, including TGF-β, Notch, and NF-κB, and EMT-promoting transcription factors, including Twist, Snail, Slug, and, Sip [42]. In the same vein, HIF-2α is also implicated in the stimulation of EMT-related factors, such as E-cadherin, LOX, CXCR4, Twist, and Zeb1 [7, 9, 42]. On the other hand, a published study showed that hypoxia induced mesenchymal to epithelial transition through the activation of HIF-2α in MSTO-211H cells, an HMM cell line [46]. Cell types and experimental conditions may be responsible for the distinct response to hypoxia with regard to the phenotypic change. Although further studies are required to determine the underlying mechanisms, EMT phenotypes under hypoxia can shed light on understanding how the hypoxic microenvironment governs the malignant progression of HMM.

## Conclusion

Like other tumors, HMM contains significant areas of hypoxia, but little information is available on the relationship between hypoxia and tumor aggressiveness. The present study illustrated the importance of hypoxia in the progression of HMM cells. Understanding the signaling pathways and molecular mechanisms affected by hypoxia may contribute to the development of therapeutic strategies targeting the microenvironmental influence on HMM biology.

## Additional files

Additional file 1: **Table S1.** The list of primers used in this study. (XLSX 10 kb)

Additional file 2: All dataset and their information about statistical analysis in the present are deposited in this Excel spreadsheets. (XLSX 24 kb)

Additional file 3: **Figure S1.** The effect of hypoxia on the abundance of HMM cells with CD44 expression. The percentage of cells with high CD44 expression is significantly higher in HMM cultured in hypoxia than those cultured in normoxia. Representative histogram of CD44 expression is presented. * *P* value < 0.05, as calculated by Student’s *t*-test. (TIF 719 kb)

## Abbreviations

Bax: Bcl-2-associated X protein
Bcl-2: B-cell lymphoma 2
Bcl-xL: B-cell lymphoma-extra large
CSCs: Cancer stem cells
EMT: Epithelial to mesenchymal transition
FITC: Fluorescence isothiocyanate
Glut-1: Glucose transporter 1
HIF-1/2α: Hypoxia-inducible factor 1 and 2 alpha
HIF-1α: Hypoxia-inducible factor 1 alpha
HIF-2α: Hypoxia-inducible factor 2 alpha
HIFα: Hypoxia-inducible factor alpha
HMM: Human malignant mesothelioma
MTT: 3-(4,5-dimethylthiazol-2-yl)-2,5-diphenyl tetrazolium bromide
Oct4: Octamer-binding transcription factor 4
PHD: Prolyl hydroxylases
PI: Propidium iodide
