# Supplementary figures and images for: Hypoxia promotes acquisition of aggressive phenotypes in human malignant mesothelioma
Source: BMC Cancer. 2018 Aug 15;18:819. doi: 10.1186/s12885-018-4720-z (PMC6094475; doi:10.1186/s12885-018-4720-z)

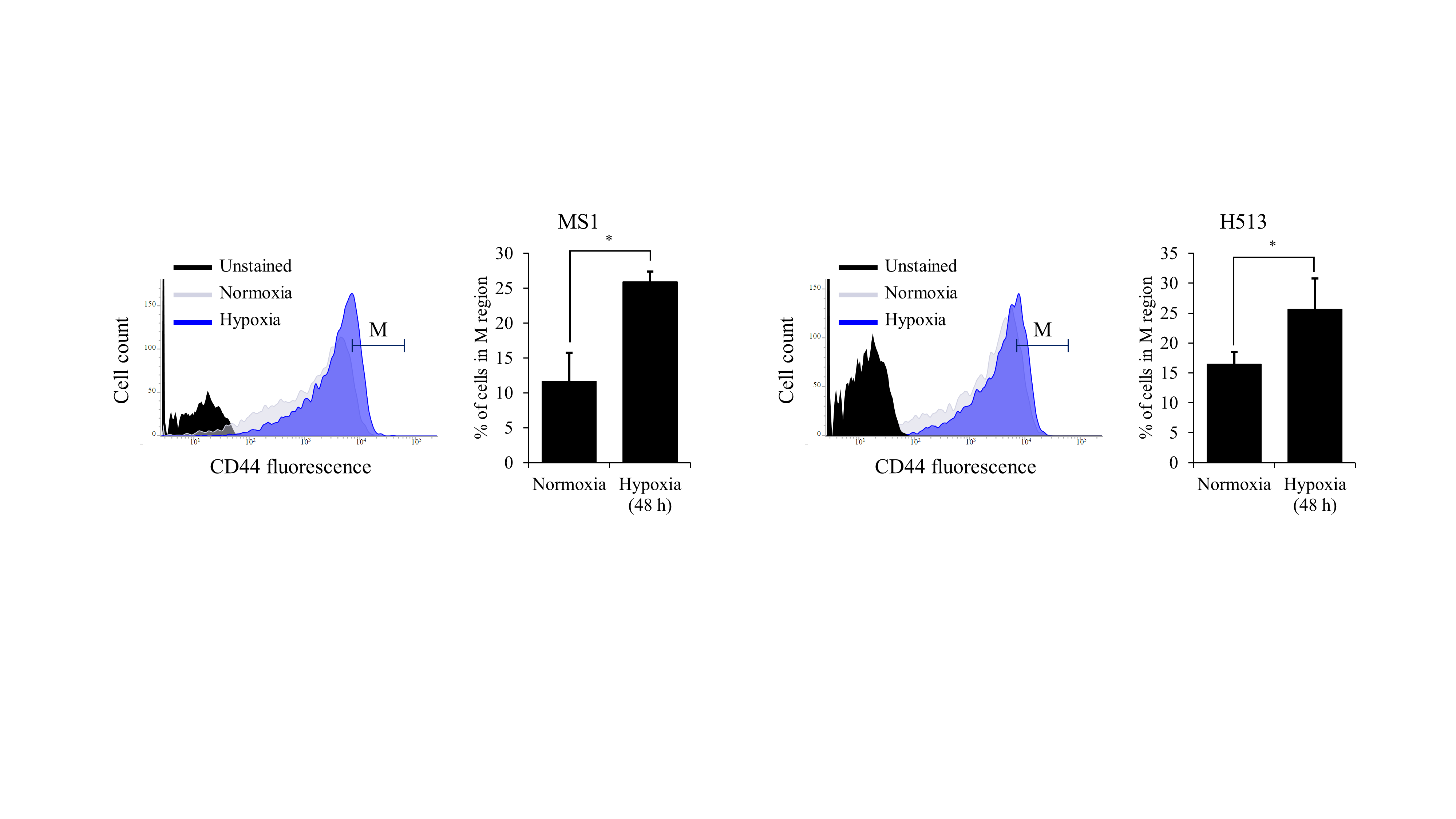

Supplement: Supplementary file 3 — Figure S1. The effect of hypoxia on the abundance of HMM cells with CD44 expression. The percentage of cells with high CD44 expression is significantly higher in HMM cultured in hypoxia than those cultured in normoxia. Representative histogram of CD44 expression is presented. * P value < 0.05, as calculated by Student’s t-test. (TIF 719 kb) [file 12885_2018_4720_MOESM3_ESM.tif]
